# An Ethnographic study of unhealthy alcohol use in a Danish Emergency Department

**DOI:** 10.1186/s13722-021-00269-z

**Published:** 2021-10-02

**Authors:** Ditte Maria Sivertsen, Ulrik Becker, Ove Andersen, Jeanette Wassar Kirk

**Affiliations:** 1 Department of Clinical Research, Copenhagen University Hospital, Hvidovre, Denmark; 2 National Institute of Public Health, University of Southern Denmark, Odense, Denmark; 3 Faculty of Health Sciences, Institute of Clinical Medicine, University of Copenhagen, Copenhagen, Denmark; 4 Department of Public Health, Nursing, Aarhus University, Aarhus, Denmark

**Keywords:** Alcohol, Context, Culture, Determinants, Emergency Department, Ethnography, Implementation, Screening

## Abstract

**Background:**

Emergency Departments (EDs) are important arenas for the detection of unhealthy substance use. Screening, Brief Intervention, and Referral to Treatment (SBIRT) for unhealthy alcohol use has been used in some ED settings with funding support from external sources. However, widespread sustained implementation is uncommon, and research aimed at understanding culture as a determinant for implementation is lacking. This study aims to explore cultural practices concerning the handling of patients with unhealthy alcohol use admitted to an ED.

**Methods:**

An ethnographic study was conducted in an ED in the Capital Region of Denmark. The data consists of participant observations of Health Care Professionals (HCPs) and semi-structured interviews with nurses. Data was collected from July 2018 to February 2020. A cultural analysis was performed by using Qualitative Content Analysis as an analytic tool.

**Results:**

150 h of observation and 11 interviews were conducted. Three themes emerged from the analysis: (1) *Setting the scene* describes how subthemes “flow,” “risky environment,” and “physical spaces and artefacts” are a part of the contextual environment of an ED, and their implications for patients with unhealthy alcohol use, such as placement in certain rooms; (2) *The encounter* presents how patients’ and HCPs’ encounters unfold in everyday practice. Subtheme “Professional differences” showcases how nurses and doctors address patients’ alcohol habits differently, and how they do not necessarily act on the information provided, due to several factors. These factors are shown in remaining sub-themes “gut-feeling vs. clinical parameters,” “ethical reasoning,” and “from compliance to zero-tolerance”; and (3) *Collective repertoires* shows how language shapes the perception of patients with unhealthy alcohol use, which may cause stigma and stereotyping. Subthemes are “occupiers” and “alcoholic or party animal?”.

**Conclusions:**

Unhealthy alcohol use in the ED is entangled in complex cultural networks. Patients with severe and easily recognizable unhealthy alcohol use—characterized by an alcohol diagnosis in the electronic medical record, intoxication, or unwanted behavior—shape the general approach and attitude to unhealthy alcohol use. Consequently, from a prevention perspective, this means that patients with less apparent unhealthy alcohol use tend to be overlooked or neglected, which calls for a systematic screening approach.

**Supplementary Information:**

The online version contains supplementary material available at 10.1186/s13722-021-00269-z.

## Background

Unhealthy alcohol use constitutes an important risk factor for public health [1] and it encompasses the spectrum from a risky use of alcohol above recommended limits to alcohol use disorders (AUD) ranging from mild to severe, if at least two out of eleven diagnostic criteria are met [2, 3]. Denmark is characterized by a liberal alcohol policy and, even though the average alcohol intake has declined over the last 20 years, there is still a substantial amount of people with a high alcohol intake, especially among young people [1, 4, 5]. In Denmark, around 20% of the adult population consume more than 14/21 (women/men) drinks per week [6]. Among patients in general hospital wards 16–26% have an alcohol consumption above this level [7–9] whereas up till 40% has been reported in Emergency Departments (EDs) [10]. However, the numbers are likely higher since self-reporting of lifestyle issues is associated with social desirability response bias [11, 12]. Since approximately 70% of the patients are discharged directly from the ED [13] and patients with a first-time hospital contact related to alcohol have a highly increased mortality [14], the ED provides a ‘window of opportunity’ to identify alcohol-related lifestyle issues [15].

Recently Danish policy makers have shown interest in early detection of patients’ unhealthy alcohol use in hospital settings, since current efforts are inadequate [16–18]. One way to detect and intervene in unhealthy alcohol use at an early stage is by using the 3-stepped SBIRT (Screening (S), Brief Intervention (BI) and Referral to Treatment (RT)) model to identify and minimize hazardous and harmful drinking developed by the World Health Organization [19–21] and funded in emergency department settings in the U.S. by Substance Abuse and Mental Health Services Administration (SAMHSA). This model has been examined in numerous studies showing varying efficacy and effectiveness in combinations of S, BI and RT in primary care, emergency department, and hospital settings [10, 22–28]; evidence for RT alone is lacking, and screening alone may have beneficial outcomes [29], or screening followed only by simple feedback or leaflets [10]. Though evidence for SBIRT in emergency department (ED) settings is not strong, one study showed that implementing a computer assisted full SBIRT program is feasible in the ED [30] and another achieved high screening rates by integrating screening in the electronic triage system [31]. Despite the potential benefits of early intervention, widespread sustained implementation of SBI efforts in ED and hospital settings is still missing, despite studies that may help facilitate this [32–35].

Interview and survey studies have examined barriers and facilitators towards the delivery of early intervention (SBI); these results have primarily reflected the individual level such as attitudes, lack of knowledge, training, personal perceptions of limited time, resources and management support [36–38]. Meanwhile, it is widely recognized that contextual and cultural factors including the collective level are important determinants that affect implementation and effectiveness [39, 40]. Context is defined as “*a set of characteristics and circumstances that consist of active and unique factors that surround the implementation effort*” [41], whereas culture is *“a pattern of meanings that are actualized in human actions and interpretations, in social institutions and in concrete, commonly available symbols, things, concepts and attitudes*” [42].

As a pre-implementation assessment, this study used an ethnographic method [43] investigating the daily context and culture in which a preventive alcohol intervention could work in an ED in order to grasp the cultural complexities and to understand the basis for uptake. By observing HCPs’ daily practices in naturally occurring situations, it is possible to gain micro-level insights of the local culture and analyze underlying patterns that showcase which factors, internal logics or collective actions could either support or prevent a successful implementation [43]. Therefore, this study explored cultural practices concerning handling of unhealthy alcohol use in an Emergency Department—how and under which circumstances is alcohol use addressed?

## Methods

### Study setting

Denmark has a public funded health care system with free access for all citizens independent of income and insurances. Hospitals often treat withdrawal symptoms, detoxification, and alcohol-related co-morbidity, while the municipalities are responsible for offering prevention and alcohol treatment. The study was conducted in an ED at a university hospital in the Capital Region of Denmark covering an area of half a million citizens. The department is divided into three units; I) A fast-track/triage area with four beds, II) An emergency room, with ten rooms for injuries and III) A medical acute ward with 29 beds. In total, approximately 190 patients are treated on a daily basis.

### Study design

This study is a qualitative exploratory ethnographic field study [44]. With this methodology it is possible to design a context-tailored intervention adapted to local practice with involvement of relevant stakeholders [45]. The study is part of a larger project, SHARE, designed as a hybrid-III implementation study [46] with a focus on implementation outcomes (e.g. acceptability) and to a lesser degree on intervention outcomes (e.g. effectiveness in reducing alcohol use).

### Ethnography

Ethnography covers participant observations and interviews and is a way to unfold culture by following health professionals in their local practice [44]. Participant observations provide knowledge on what is deemed important by HCPs [47]. Further, their daily practices display when, why and under which conditions a preventive intervention, such as SBI, could be integrated. Interviewing provides access to the individual’s interpretation of the studied social world and the purpose is to obtain a diversity rather than consistency in responses [48].

### Data collection

#### Observations

Participant observations were conducted between July 2018 and February 2019 [44]. The first author (DMS) was present on both weekdays and weekends in day-, evening- and night shifts. The empirical object was *unhealthy alcohol use* [2]. Since we have a preventive focus in this study, we were particularly interested in patients who might be hospitalized with a different diagnosis, but with alcohol use ranging from drinking above recommended limits to meeting diagnostic criteria for AUD, *if* they had been screened [2]. We exploratively examined physical spaces, interactions, and artefacts related to HCPs’ handling of patients’ alcohol use [49]. A variety of situations were observed e.g. receiving new patients, ward rounds, handovers, journal recording of newly admitted patients, daily update meetings by the electronic boards and daily care. Between 1 and 3 HCPs with varying professional experience were followed per day. We chose a passive (common) to moderate (infrequent) observer role [50], since a more participatory role would have required ED experience and special competencies. Situations where the researcher would switch degree of participation were in service-related interactions with patients, such as getting a glass of water or a blanket. Since the topic of alcohol is sensitive, the observer had a focus on being present in the moment, without creating distraction or distrust [44]. All situations were assessed with *situational ethics* [51] and reflections of the observer role was discussed with co-author JWK continuously. Brief notes were made during observations and extended field notes were written in a Word document after every observation day.

#### Interviews

Semi-structured interviews with ED nurses were conducted in January and February 2020 by DMS [52]. The interviews were held in an office near the ED during working hours. Five out of eleven nurses had been followed during observations. Interviews lasted from 20 to 60 min (mean: 34 min). Interview guides were developed, tested, and adjusted by DMS based on the in-depth analysis from the observations and were approved by the author group. The interview questions focused on the main findings from the observations that needed further elaboration (Additional file 1). Interviews were transcribed verbatim by DMS and a research colleague not otherwise involved in the project.

### Ethics

The project was performed according to the Helsinki Declaration [53] and approved by the Danish Data Protection Agency (VD-2018-229, I-Suite 6471). Results are presented in line with *Standards for Reporting Qualitative Research* (SRQR) [54].

### Data analyses

We conducted a *cultural analysis*, which is a synthesis and constant interplay of testing theoretical and analytical concepts between the empirical object and the analytical field [47]. The analytic process was 2-stepped: first, as an inductive analytic tool, we used Qualitative Content Analysis to identify common threads across the data set [55]. Analysis and data interpretations were discussed concurrently with JWK. To strengthen validity further, all authors read the coded data material and developed themes in an iterative process, and then reviewed the need for further refinement (Table 1). Interviews were based on findings from the observations and the same analysis process was conducted with this data material. If meaning units could be understood in terms of two different themes, they were cross-indexed. Next, as an implicit part of the cultural analysis, we theorized and interpreted findings from the qualitative content analysis, which is presented in the discussion part of this paper.

**Table 1:** Example of the analytic process of qualitative content analysis within theme 2 “The encounter”

Meaning unit	Condensed: description close to the text (manifest)	Condensed: interpretation of the underlying meaning (latent)	Subtheme	Theme
“Following the present guidelines, we ask about smoking and alcohol, but you don’t do anything about it No action is taken on the information provided” (Doctor, field notes, day 28)	The doctors ask about alcohol and smoking habits but do not act on the info	Health policy requires lifestyle issues to be addressed, practice follows requirements without further action	Professional differences	The encounter
”It’s also a matter of subjective assessment (…) I’m a little suspicious and think… I look at their clothes and they are maybe a little untidy and I wonder if their home situation is OK” (Interview 7)	Patients appearing unkempt with an unclear home situation may give rise to suspicion of an unhealthy alcohol use	Clinical assessment of an unhealthy alcohol use is (among other things) based on both visual appearances and patients’ capabilities	Gut-feeling vs. clinical parameters	The encounter
"If they initiated a dialogue, they should also finish it, which they did not feel they had time for It would be unethical to start something they could not finish” (field notes, day 7)	It is unethical to start a talk you cannot finish	Time is a barrier for asking about alcohol and is used as an ethical argument, that governs the patient encounter	Ethical reasoning	The encounter
"An alarm sounds […] Secretary said that a ‘drunk’ man had punched a female patient in the face So, they had ‘kicked’ him out” (field notes, day 27)	The alarm went off. An intoxicated man hit a fellow patient and has been thrown out of the hospital	A safe hospital stay is a top priority. Patients who cross the line of accepted behavior must leave	From compliance to zero tolerance	The encounter

## Results

In total, 150 h of participant observations of HCPs obtained in 38 observation days and 11 interviews with nursing staff was included in this study. Of the 11 interviews, 10 participants were women and the mean work experience in the ED was 5.8 years (range 1–16 years) and the general experience was 11.4 years (range 5–26 years).

Three themes emerged; *Setting the scene*, *The encounter* and *Collective repertoires* (Table 2).

**Table 2:** An overview of themes and subthemes

	Themes	Subthemes
1	Setting the scene	Patient flow in acute medicine
		A risky environment
		Physical spaces and artefacts
2	The encounter: addressing patients’ alcohol use	Professional differences
		Gut-feeling vs. clinical parameters
		Ethical reasoning
		From compliance to zero tolerance
3	Collective repertoires	”*Occupiers*”
		“*Alcoholic*” or “*party animal*”?

### Theme 1

#### Setting the scene

This theme concerns the wider context of the ED related to patients with unhealthy alcohol use. Maintaining flow is a driving force in the ED. Acute conditions and saving lives are core tasks. However, many admitted patients have chronic illnesses, alcohol or substance use. Keeping constant flow is a mindset that permeates both nurses’ and doctors’ approach to the patients and triage plays an important part in this process:

*We take care of two things here: detoxification and withdrawal symptoms. When people are severely affected consciously, we must of course rule out that nothing else is wrong. When that’s clarified, they must be detoxed and then sent home (Field notes, Doctor, day 16)*

The HCPs faces the risk of physical—or verbal outbursts from patients (usually under influence of substances). Assault alarms hanging on the walls reflect these threats, however they are not used by all, since the HCPs do not wish to appear offensive. Mainly secretaries and HCPs during evening or night shifts use them. Also, most of the HCPs cover their identity with stickers on their name tags as an adaption to a risky environment:

*This is a patient group that we have to accept, but we always associate it with a feeling of unease and instability in the ward … we know them, you see (Interview, Nurse 3)*

One patient room is used frequently for patients with unhealthy alcohol use. The room, called *the observation room*, is located furthest away, and does not have monitoring opportunities, since it is meant for short observations of minor conditions. This room also has a nickname [da. *slyngelstuen*], which means the room for villains or rogues. On several occasions, the HCPs articulates that by placing patients here they are aware that they stigmatize. Nonetheless, they feel ambivalent about doing it and are aware that it should not be a common practice. Yet, it is accepted to place certain patients in that room because these patients sometimes cause insecurity among the other patients:

*I also think that's why we sometimes isolate them in the observation room, even though it was never the intention […].But when older ladies grab ones’ hand and says: “I'm scared to lay in the bed next to him and now he's doing that … now he’s saying this… I don’t want to be here”. Understandably, it creates insecurity (Interview, Nurse 4)*

Therefore, patients with known unhealthy alcohol use are often placed in the same physical room as a precaution. The term *cohort isolation*, which is normally used for infectious diseases, is used among HCPs for patients with alcohol or substance use. The isolation sometimes causes negative consequences such as quarrels or assault between patients, but patients can also make alliances. The following paragraph shows a culturally embedded behavior, where placement of the patients is seemingly a well-known practice to external collaborators:

*On the electronic board I can see that they are expecting the arrival of a patient who has been drinking too much. It doesn’t take long before two paramedics enters the office. They tell the coordinating nurse: “we are here with [name] and he has been drinking a bottle of vodka. […] we’ve placed him in the observation room”. The coordinating nurse says: “why did you put him there without asking?” and the paramedic says: “that’s where they usually are, so we just assumed”. The coordinating nurse shrug her shoulders. The patient stays in that room for the rest of my shift. (Field notes, day 14)*

According to the HCPs, the physical surroundings and the lack of privacy does not support having confidential conversations about alcohol. However, many other types of personal conversations and sensory experiences that fellow patients can see, hear, or smell *do* take place in the room. The HCPs explain that they usually place patients with unhealthy alcohol use together to protect or shield other patients. The consideration for other patients legitimizes this behavior where the HCPs act in a stigmatizing way towards a certain group of patients:

*It is a misunderstood consideration, because we care for the other patients, by treating one group very badly by isolating them from the rest. We tend to… those who smell we put there, but an old lady can also smell badly, because she doesn’t shower anymore. But we would never consider putting her in that room (Interview, Nurse 8)*

During observations, patients with a newly discovered unhealthy alcohol use were also placed here, even though they did not show any unwanted or offensive behavior:

*A man in his thirties entered the ED with ankle-pain after a fall. While waiting for a physiotherapist, he suddenly gets withdrawal symptoms, and they realise he needs a longer admission and is placed in the observation room (Field notes, day 4)*

An artefact with a central role is the electronic board. On the board HCPs gains an overview of expected and admitted patients. After every name a clock shows length of stay. Diagnosis at admittance and other relevant keywords are also written here:

*We enter the office and the doctor shows me the board and says: ”Look, there are no alcoholics today, totally empty”. I tell him that I’m particularly interested in those patients who might come in under a different diagnosis with an undiscovered high alcohol intake [and thereby potentially all patients]. ”They’re usually here” he says and point at the observation room, ”There are none today”. (Field notes, day 24)*

This reflects how the board as an artefact governs HCPs focus and prioritization. No visual diagnosis or words that indicate an alcohol use appear on the board which becomes a contributing factor to whether patients with unhealthy alcohol use are detected or not, and if it is acted upon.

### Theme 2

#### The encounter: addressing patients’ alcohol use

This theme concerns the encounters HCPs have with patients when receiving them at admittance, during rounds, and in daily care routines. The theme illuminates how, why, and under which circumstances unhealthy alcohol use is addressed in clinical practice.

If the patient is admitted with either an alcohol-related diagnosis or an injury while smelling of alcohol or being visibly intoxicated, the nurses are prone to ask about alcohol. They might ask “*were you drunk when it happened*?” or ask about the consumed amount today “*how much did you drink tonight*?”, rather than ask standardized screening questions about the patient’s usual intake. Often, questions relate to the actual moment to understand the current situation and to provide the necessary treatment instantly. Based on experiences of patients refusing, denying, or lying about their intake, nurses usually feel that it is more legitimate to ask if they relate the question to the actual treatment and make their professional secrecy very clear. If the alcohol intake is not obvious, it may not occur to them:

*If he is tossing and turning in bed, of course, I try to find out what’s wrong... If he's just a nice guy with an excessive alcohol intake, I might not notice it… it doesn’t strike me (Interview, Nurse 5)*

Whether the nurses ask questions or not is often determined by subjective assessments along with clinical symptoms like a blood sample, perspiration, tremor, or high pulse. Although clear indicators do not necessarily mean that alcohol is addressed:

*He enters carrying a clinking bag [with beer bottles]. He is in a cheerful mood and jokes a lot […] The nurse asks what has happened […] She cleans the wound on the big toe, applies a bandage, and gives him a tetanus vaccination. She doesn’t ask about alcohol (Field notes, day 7)*

Several HCPs mention a *sense* or a *gut feeling* about unhealthy alcohol use. Visual appearances like personal hygiene, “*worn or ravaged look*”, flushing of the skin, and conspicuous or “*odd behavior*” typically make them suspect an alcohol use. Several mention that if the unhealthy alcohol use is not described during a previous hospital stay, the chances of recognizing an unhealthy use is low, unless the patient fits some of the descriptions mentioned above.

Most HCPs do not wish to appear judgmental or paternalistic. It is expressed in different ways; ethical arguments are used to explain why unhealthy alcohol use is not addressed in all cases. One doctor finds it unethical to burden the patient with questions, that are not directly connected to the problem that causes the admission. Others explain or justify that they will not start a conversation, which potentially opens “*Pandora’s box”*, if they cannot finish it. On several occasions, when intoxicated patients are admitted *with* a condition linked directly to alcohol, e.g. an injury, nurses ask superficially, but avoid going further into the patient’s history of alcohol use by normalizing drinking culture and being non-judgmental:

*The nurse asks if he has been drinking and he says yes. “That’s ok, you’re allowed to”, says the nurse. (Field notes, day 33)*

Journal recording is often a task assigned to junior doctors; therefore, it is common for them to address alcohol use while examining the patient. Patterns in the empirical data showed that the task is performed in a non-systematic manner where each individual chooses how to phrase questions, which actions to take and *if* a referral to a specialist is needed. As it is a mandatory task mostly carried out by the junior doctors, the senior doctors can attend to their main expertise:

*The senior doctor says he is not interested in alcohol. He is interested in the heart and lungs. Typically, the junior doctors handle “the alcoholics” (Field notes, day 16).*

A persistent mantra among HCPs is, that the hospital should be a safe place. All patients are entitled to feel safe here. This statement means that *if* a patient causes insecurity among HCPs and fellow patients, the issue is addressed and dealt with. In severe cases HCPs operate a zero-tolerance policy and the patient is told to leave the department immediately. Situations that causes zero tolerance are e.g. sexual assault on a fellow patient and unmotivated violence between fellow patients. The unwanted behavior of certain patients with unhealthy alcohol use lead to a saturation among HCPs and many expressed a frequent strategy is to become either indifferent or even cynical at times:

*She refers to a patient who is a frequent visitor in the department, and she says: "I already know what he will say and what he wants". So, we will probably give people like him a lower priority, because we have already tried EVERYTHING. (Field notes, day 11)*

### Theme 3

#### Collective repertoires

This theme illuminates how language is central in shaping the collective perception of unhealthy alcohol use in the ED. A repertoire is repeated phrases or expressions that a person habitually uses. In relation to alcohol, HCPs highlight a specific group of patients, even though the empirical object is unhealthy alcohol use in broad terms. This group of patients are very present in the data material and in the following they are called the empirical term “*occupiers*”:

*I think it’s a difficult group to deal with. The group is challenging and require a disproportionate amount of time and energy […] They occupy a great deal of resources with their presence! (Interview, Nurse 2)*

This empirical term covers patients with (often a combination of the following) severe alcohol or substance use, psychiatric disorders, and homelessness. HCPs expresses that this group of patients typically demands a disproportionate amount of energy, resources, and patience. Their life situations are often overwhelming and tragic. Several HCPs tell that it is hard to take it all in, that they must “*toughen up*” to work in this environment. they explain that they build up a filter and a kind of “*immunity*” to deal with the hopelessness and powerlessness connected to this patient group. A consequence is to lose interest:

*We lose interest in these patients because we feel there is no progress. We don’t feel that what we do helps. Not at all. I just feel that we are some sort of storage facility. (Interview, Nurse 3)*

Viewing the ED as “*a place for storage*” contradicts the mindset of *flow* and, furthermore, no positive development occurs. From this perspective, the patient occupies a bed that could be used for someone else. Questions of ethical and ontological character emerge repeatedly in daily practice situations: “*Are alcohol problems a ‘real’ disease?*”, “*Is it self-inflicted?*” and “*What constitutes a real patient and thereby entitles somebody to treatment?*”. Even though it is sometimes said in a joking manner, a priority between patients is made every day in terms of triage or instant worsening of an acute condition, which affects HCPs’ attentiveness towards other health care problems. It is a common experience among HCPs that they see *many* patients with a severe unhealthy alcohol use daily and some wonder how many patients with a less severe use there actually are in the ED:

*We have… I allow myself to call them the poorest of the poor. We rarely admit the ones you mention [patients with an alcohol use, admitted with another diagnosis] […] It's mostly the hardcore types, who in my opinion are not interested in getting any help. (Interview, Nurse 9)*

Another distinction frequently used among HCPs is whether a patient admitted for any condition related to alcohol is an “*alcoholic*” or a “*party animal*”. According to HCPs “*The alcoholic is characterized by a long-term or chronic dependency*” and is often accompanied by various severe social problems. Whereas a pattern in the data discloses that in HCPs’ opinion, “*the party animals*” are “*ordinary people*” in control of their lives during the week, who just have a fun night and are usually in the ED Fridays and Saturdays. What both labels have in common is, that these patients are admitted with either a visible alcohol-related diagnosis on the electronic board, an injury or visual intoxication. Subjective assessments and blurred lines between the two labels occurred in practice:

*I asked the nurse of the patient who had been admitted intoxicated yesterday [if I could follow her]. She said, she had already talked to him and he was being discharged. She said: ‘He is not an alcoholic. I just told him to go home and drink some coke’ (Field notes, day 2)*

An interesting term found somewhere in between*”party animal*” and”*alcoholic*” is”*a first-time detoxer*”; a person admitted for detoxification for the first time. This classification has more hope connected to it than”*alcoholic*” since some of the HCPs express that, they feel, they can actually *do something* to help this person in the right direction.

## Discussion

The main results show that contextual factors such as artefacts, physical spaces, collective actions and attitudes in the encounter and collective repertoires point HCPs’ attention in a certain direction. These factors contribute to HCPs’ assessment of who drinks too much and who does not, resulting in mainly patients with severe unhealthy alcohol use are recognized. The term *messy object* which is defined as an “*interpretatively complex object of multiple perspectives”*, and further as being *fuzzy*, *moving* and *a shape-shifting target* can be used to interpret our findings [56, 57]. It originates from a study of patients with Alcoholic Liver Disease (ALD) showcasing that in clinical practice ALD also covers other classified diseases such as liver disease, cirrhosis or AUD [56, 57]. Implicitly in a messy object there are two interrelated and co-existing terms: The *presence* of any object appears in a network of relations and always implies an *absence* of something else and vice versa [57]. In the results, it appears that whether an unhealthy alcohol use is *absent* or *present* depends on the complex relational network it is entangled in. As an example, the empirical data shows how a group of patients with less apparent unhealthy alcohol use “disappears” in the ED (*absence*) since they are not characterized by diagnosis on the electronic boards, visibly intoxicated or displaying a certain behavior. In contrast, what becomes very *present* are the patients with visible unhealthy alcohol use and “*the occupiers*” that demands a certain amount of time, energy and resources from HCPs. This *presence* of substantial unhealthy alcohol use tends to shape the culture in the ED—how to act and speak in relation to *all* patients with unhealthy alcohol use. This causes an *absence* of focus on less apparent alcohol use, which consequently recede into the background or in some cases are entirely neglected. Our results emphasize that patients with unhealthy alcohol use can easily be overlooked and underlines the need for a universal screening in the ED population in contrast to symptom-specific screening [58, 59]. This is supported by a meta-analysis on clinical judgement, presenting that hospital staff is able to identify AUDs in 52.4% of the cases [60]. Further, results are supported by studies describing HCPs’ reluctancy to ask if there are no obvious or visual signs of alcohol use [61–63]. If a structured approach to alcohol use is not implemented, there is a risk of unhealthy alcohol use being left undiscovered.

Unhealthy alcohol use has been perceived as a continuum from no risk to severe AUDs for decades [64]. Nevertheless, by looking at the results, it appears that a dichotomous divide exists, when HCPs assess patients to either have a “problem” or not. This distinction between having an unhealthy use or not, can be explained by the coherent concepts of social classification; *lumping* and *splitting* [65]. *Lumping* is when people cognitively group somewhat similar things or people even though there may be differences between them [65]. In *splitting*, the opposite happens; differences are enforced which creates a gap between the cognitive groupings [65]. In the results, it is shown how the “*occupiers*” shape the general approach to alcohol in the ED, which is a cultural pattern of *lumping*, where patients’ similarities may be more occurrent than their differences to the HCPs, which potentially can cause stereotyping and thereby stigmatization [65]. In relation to stigma, how HCPs talk (collective repertoires) govern their practices, which over time can cause a construction of cultural truisms. If these repertoires are reproduced continuously in relation to alcohol use, there is a risk of perceiving the patients as fixed stereotypes.

Another important contextual factor is *flow*. In literature characterized as *flow culture*, where HCPs actions leading towards patient flow are rewarded [66]. Hence, screenings and guidelines supporting flow are more likely to be accepted by HCPs, in opposition to those not supporting flow (*flow-stoppers*) [67]. Based on our findings, it seems that both “*the occupiers*” and other patients with unhealthy alcohol use may be perceived as *flow-stoppers* in terms of either demanding energy and resources from the HCPs, or they are not given the attention of preventive actions, since preventive work with a goal beyond ED admission is not supportive of flow. Thus, screening for unhealthy alcohol use that seemingly does not support the flow can be challenging to implement, unless it facilitates flow. Considering the existing *flow culture* (and its underlying structures and motivators) is essential if the aim is to implement a sustainable preventive procedure in ED settings.

The knowledge from this study can be used to guide future implementation efforts of alcohol preventive interventions. In alcohol research, tailoring and matching implementation strategies and interventions has been advocated for [32], and efforts towards this have been done in practice-oriented studies [68, 69]. Our results are primarily aimed at structural and behavioral determinants, which points to the importance of planning-, restructuring-, quality management- and educating strategies [70]. However, implementing such an initiative is most likely a top-down decision. If researchers and policy makers require a sustainable solution, they need to understand and consider the cultural practices that shape the HCPs’ actions.

## Limitations

Screening was not performed during observations, since it was not standard practice and would thereby not reflect HCPs point of view. We strived for diversity in participant sampling. However, most had several years of working experience. The results may have been different if newly educated nurses had been interviewed, albeit a minimum of two years working experience is required to get employment in an ED. In addition, experienced staff are role models shaping and influencing the culture. The interviews were conducted 12 months after the observations ended, which may have caused a risk of recall bias. However, an in-depth analysis of fieldnotes had to be carried out first to inform the interview guide. Additionally, findings from observations were confirmed in interviews. The first author (DMS) was positioned as an *outsider*, which implied continuous negotiation of access thoroughly described in fieldnotes for transparency [71]. Fieldnotes and interpretations were discussed among the authors to ensure trustworthiness and validity. The credibility was enhanced by conducting fieldwork in all shifts throughout the week and by following many different health professionals.

## Conclusion

This study demonstrates that the cultural practices of handling patients with unhealthy alcohol use in an ED are related to an array of challenges, besides the well-known barriers and facilitators that can affect future implementation. Physical spaces and artefacts, collective actions, encounters and repertories form a culture that influences how and when patients’ unhealthy alcohol use is addressed. Culturally, unhealthy alcohol use is entangled in a complex relational network of *presences* and *absences* that influences the recognition of a potential unhealthy alcohol use. Implications of these findings constitute a paradox in a preventive perspective. Even though there is a political will to prevent unhealthy alcohol use by earlier identification, the clinical practice is characterized by a culture in which the severity of unhealthy alcohol use in certain patients governs the approach to unhealthy alcohol use in general, neglecting those patients more easily accessible to prevention.

## Supplementary Information

**Additional file 1: Table S1**. Examples of interview questions based on observations

## Data Availability

The dataset generated and analyzed in the current study is not publicly available due to potentially identifying or sensitive information in fieldnotes and interviews. However, upon reasonable request all data is available from the corresponding author, after taking all necessary precautions to protect the participating ward and participants’ confidentiality.

## Abbreviations

AUD: Alcohol Use Disorder
ED: Emergency Department
HCPs: Health Care Professionals (Nurses and doctors)
SBIRT: Screening, Brief Intervention and Referral to Treatment
