# Supplementary material for: An Ethnographic study of unhealthy alcohol use in a Danish Emergency Department
Source: Addict Sci Clin Pract. 2021 Oct 2;16:60. doi: 10.1186/s13722-021-00269-z (PMC8487327; doi:10.1186/s13722-021-00269-z)
Supplement: Supplementary file 1 — Additional file 1: Table S1. Examples of interview questions based on observations [file 13722_2021_269_MOESM1_ESM.docx]

# Additional file

Additional file 1. Table. Examples of interview questions based on observations.

| 1 | In your opinion, what are indicators of a high alcohol consumption? |
| --- | --- |
| 2 | How do you feel about addressing a patients’ alcohol habits? |
| 3 | Do you ask all your patients about their alcohol consumption? How do you ask? If not, what are your considerations for not asking? |
| 4 | If the patient reports a high intake of alcohol, then what do you do with the knowledge you’ve gained? |
| 5 | Can you describe which opportunities you can offer patients with alcohol problems? |
| 6 | During observations some staff expressed, that stigmatization occasionally happens in the Emergency department. What do you think? Have you experienced any situations where stigma occurred? |
